# Determinants influencing health-promoting behaviors in individuals at high risks of stroke: a cross-sectional study

**DOI:** 10.3389/fpubh.2024.1323277

**Published:** 2024-06-07

**Authors:** Mengxia Chen, Mengdi Wang, Mengting Qiao, Xiaorong Huang, Dongmei Li, Longjuan Yu, Lifen Gan, Wenyao Chen, Yanqiu Weng, Jingwen Zhang, Bing Yu, Jianmin Liu, Lingjuan Zhang

**Affiliations:** ^1^Education and Scientific Research Department of Clinical Nursing, Changhai Hospital, Shanghai, China; ^2^Neurovascular Center, Changhai Hospital, Shanghai, China; ^3^Shanghai Quality Control Center of Geriatric Care, Shanghai, China; ^4^Key Laboratory of Geriatric Long-term Care (Naval Medical University), Ministry of Education, Shanghai, China

**Keywords:** the high-risk population of stroke, health behaviors, health beliefs, stroke knowledge, social support

## Abstract

**Background:**

Quit smoking, moderate drinking, exercise, and healthy eating habits are all known to decrease the risk of stroke. As a result, understanding the health behaviors of high risk groups for stroke is crucial. Health behavior is influenced by knowledge, social environment, and health beliefs. However, little research has been done on these relationships. For a better grasp of the relationships mentioned above, consider using the COM-B model (capability, opportunity, motivation, and behavior). The purpose of this study was to investigate the variables related to health behavior and to test the mediating effect of health beliefs.

**Methods:**

The cross-sectional study was carried out at a physical examination center of a tertiary hospital in Shanghai, China. 986 high-risk populations of stroke have been tested using the Health Behavior Scale (HBS-SP), Stroke Knowledge Questionnaire (SKQ), Health Beliefs Questionnaire (HBS), and Multidimensional Scale of Perceived Social Support (MSPSS). The structural equation modeling was used in this study.

**Results:**

The scores for MSPSS, SKQ, HBS, and HBS-SP were 60.64 ± 13.72, 26.60 ± 9.77, 157.71 ± 34.34, and 2.46 ± 0.41, respectively. The revised model fits well (approximate root mean square error = 0.042; comparative fit index = 0.946). The health behavior was obviously and positively correlated to social Support, stroke knowledge, and health beliefs. Moreover, health belief has a mediating effect on the relation of social support, stroke knowledge, and health behavior.

**Conclusion:**

Chinese high risk groups for stroke have a mediate level of health behaviors. Factors associated with health behaviors are knowledge of stroke, health beliefs, and social support. The COM-B-based model can be used to explain the health behavior of individuals at risk of stroke and to guide the formulation of effective health management programs.

## Introduction

With more than 13 million strokes, over 2 million new cases, and an additional 23.9 million transient ischemic attacks (TIAs) per year (1, 2), stroke is the main cause of mortality and adult disability in China (3), and the economic cost of its treatment and out-of-hospital care is particularly high. Consequently, stroke places enormous pressure on patients, families, society, and medical systems (4). As part of the national noncommunicable diseases (NCDs) work, the Chinese government finally established the stroke screening and intervention project for high risk groups in 2011, and by 2016, 6 million people in 31 provinces have been screened for vascular risk factors (1). As of December 2019, the Brain Prevention Committee of the National Health Commission had licensed 30 stroke demonstration centers, 466 stroke centers, 181 comprehensive stroke prevention centers, and 717 stroke prevention centers (5).

Most of the burden of stroke can be attributed to changeable risk factors, and epidemiological studies show that these factors are related to the occurrence of stroke (6, 7). The main risk factor for stroke is hypertension (8). Additionally, research has shown that smoking significantly raises the relative risk of ischemic stroke by 90% (9, 10), making it an independent and significant risk factor. Diabetes, which can more than double the risk of stroke (11), is another independent risk factor for stroke. Atrial fibrillation and other types of heart disease may also increase the risk of stroke (12) and dyslipidemia is significantly associated with stroke (13). Increasing daily physical exercise can reduce the risk of stroke (14, 15). There is also a positive correlation between stroke and obesity.

Health behavior is an effective and practical way to prevent stroke (16, 17) and is defined as a range of overt behavior patterns, actions, and habits that relate to health maintenance, health restoration, and health improvement (18), which possibly reduces stroke risks by as much as 80% (19), and stroke incidence rates by as much as 50% (20). Numerous researchers have extensively examined the interplay between knowledge and belief and an individual’s understanding of a specific event is influenced by the information they acquire, whereas beliefs are rooted in the knowledge perceived by the individual (21). Previous studies show that stroke patients with more knowledge, strong health beliefs, and social support will greatly improve their health behavior (22, 23). Moreover, social interaction also plays a critical role in the outcome of stroke survivors and social support can also increase health belief which refers to an individual’s belief in his or her capability to execute health behaviors necessary to achieve health-related outcomes (24).

**Figure 1:**
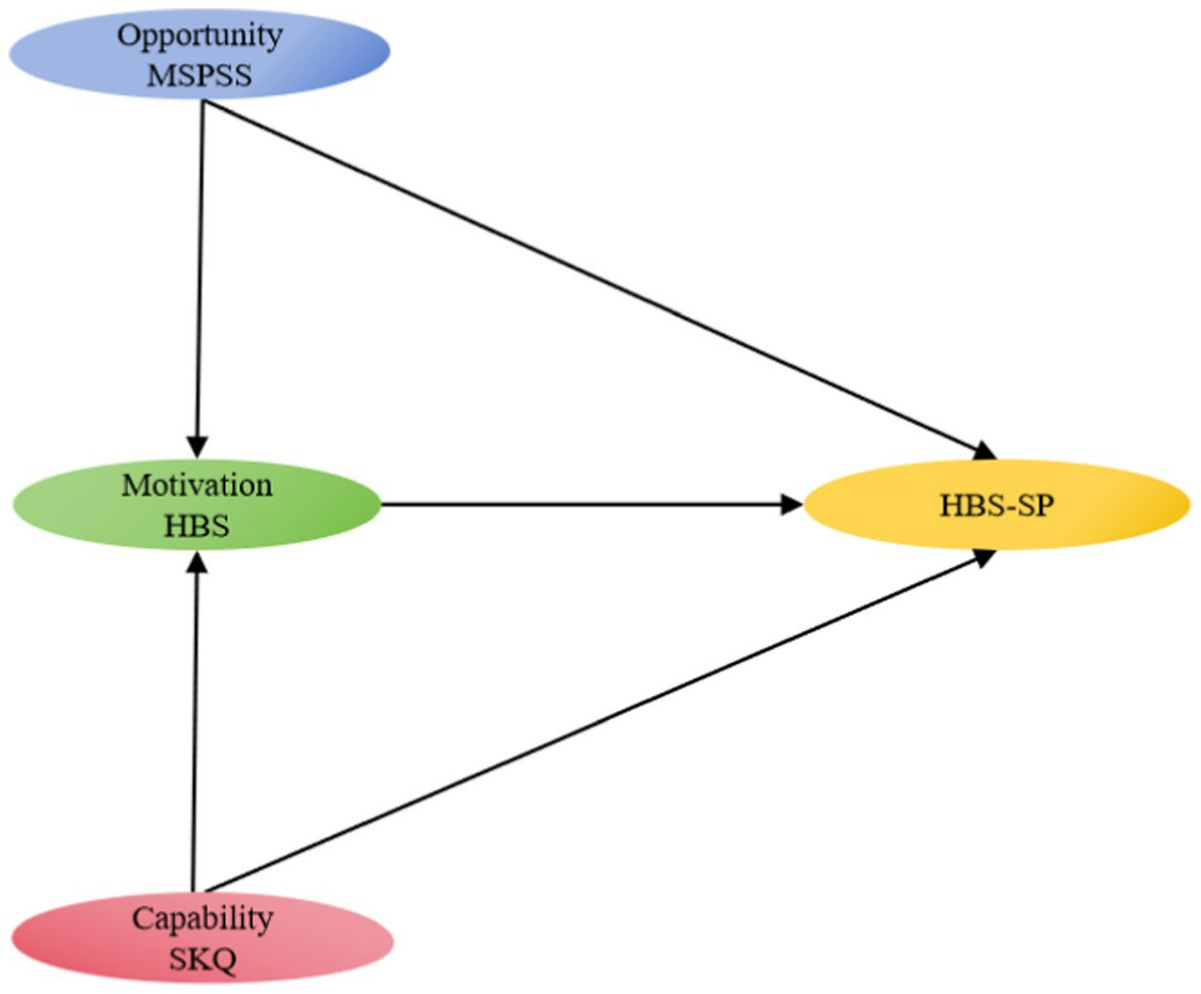
Conceptual model of this study.

However, current primary stroke prevention strategies for high-risk groups (19) fail to actively prevent most high-risk groups because of the lack of personal motivation to control risk factors (25). Individuals at risk of stroke rarely adhere to suggested lifestyle changes, from unhealthy lifestyles to health-promoting behaviors (26). Most studies on the factors that influence health behavior have rarely been influenced by a theoretical framework. As a result, employing behavioral models to identify health behaviors may provide important insights into efficient interventions to enhance health-promoting behaviors. The capability opportunity motivation and behavior (COM-B) model is a model for behavior change (27). According to the theory, behavior is produced by the interaction of capability (psychological or physical ability, such as knowledge), opportunity (physical and social environment, such as environmental resources and social impacts), and motivation (reflective and automatic mechanisms, such as self-efficacy and emotion) (28). The relationship between capability, opportunity, and conduct is mediated by motivation. This framework can help researchers diagnose what needs to be changed to achieve the desired behavior, hence assisting in the design of behavior change interventions (28). The COM-B has demonstrated substantial explanatory power for health behaviors in earlier studies (28, 29). However, only a limited amount of studies have determined the paths using structural equation modeling (SEM). Therefore, in our study, we regard capability as knowledge of stroke, opportunity as social support, motivation as health beliefs, and behavior as a healthy lifestyle. To examine the relationships between the aforementioned variables, we utilized SEM. The theoretical model is shown in Figure 1.

Based on the above discussion, we propose the following hypotheses:

*H1:* Social support, stroke knowledge, and health beliefs significantly affect the health behaviors of high-risk individuals with stroke.

*H2:* Health beliefs mediate the relationship between social support, stroke knowledge, and health behaviors.

Therefore, this research focuses on high-risk groups of stroke and conducts an in-depth review of their health behaviors. On the one hand, this study facilitates a deeper understanding of the relationship between social support, stroke knowledge, health beliefs, and health behavior while examining the possible channels through which the above variables affect health behaviors among high-risk groups from the comprehensive COM-B theory. On the other hand, to better understand the influence of beliefs, this study included health beliefs as a mediating variable. This study fills in the gaps left by earlier research and offers a solid foundation for more effective guidance aimed at enhancing the health behaviors of high-risk groups and reducing the morbidity of stroke.

## Methods

### Study design and participants

Convenience sampling and a cross-sectional design were used in this investigation. In total, 986 individuals from our hospital’s physical examination center engaged in this study. The inclusion criteria for patients in this research were (1) people aged ≥ 18 years old; (2) high-risk populations of stroke; and (3) voluntarily participating in this study. Our definition of high-risk populations of stroke in this study included subjects with high-risk factors, but no cardiovascular events. The risk of stroke was assessed according to the Chinese Stroke Screening and Prevention Program, people with three or more of the following risk factors are classified as high-risk stroke population: hypertension, hyperlipidemia, diabetes, current smoking, physical inactivity, atrial fibrillation, obese or overweight (BM ≥ 26 kg/m2), family history of stroke (30). The BMI was computed as weight/height2 (kg/m2). Individuals with communication difficulties due to severe physical or mental illnesses, serious illnesses such as malignant tumors, dementia, and schizophrenia, and disabilities were excluded. According to the requirements of the sample size of the structural equation model, the number of samples should be 10 to 20 times the number of observed variables. A total of 32 observed variables were included in the structural equation modeling in this study. The theoretical sample size is 320 to 640 people. The actual sample size in this study was 986, the effective sample size was 960, and it also met the minimum requirement of 200 people for the structural equation model sample (31).

### Instruments and measurements

#### Demographic and clinical information

Sociodemographic information included age, gender, education, marriage status, current smoking, and physical inactivity. Clinical information included hypertension, dyslipidemia, diabetes, obesity or overweight, atrial fibrillation, and a family history of stroke. These data were obtained through a comprehensive and self-made questionnaire.

#### The Chinese version of the multidimensional scale of perceived social support (MSPSS)

MSPSS was designed by Zimet et al. (32). MSPSS is a 12-item questionnaire that assesses subjective perceptions of the adequacy of social support from family, friends, and important others (romantic partners, etc.). Participants rated these questions on the Likert scale on a scale of 1 to 7, with a score of 1 as very strong disagreement and 7 as very strong agreement. Summary scores range from 12 (lowest) to 84 (highest level of social support). The Chinese version of the MSPSS has good reliability and validity in the Chinese population (33, 34). In the study, the Cronbach’s α of MSPSS was 0.979.

#### The Chinese version of the stroke knowledge questionnaire (SKQ)

SKQ is a 40-item questionnaire to evaluate personal knowledge about stroke, developed by Yao (35). SKQ has 6 dimensions: premonitory symptoms (7 items), emergency treatment (4 items), risk factors (12 items), drug safety (4 items), mode of behavior (10 items), and rehabilitation (3 items). The answer to each item gets one point and the correct answer gets one point, the wrong answer or unknown answer gets zero. Each participant’s project scores add up, ranging from 0 to 40. The higher the score, the higher the understanding of stroke. The scale is widely used in China and has good reliability and validity (36–38). In the study, the Cronbach’s α of SKQ was 0.938.

#### The Chinese version of the health beliefs scale (HBS)

HBS is a 48-item questionnaire to assess health beliefs, which was suitable for Chinese populations and was developed by Ji and Yang (39). There are 48 items in 5 dimensions, including personal health belief (10 items), feel the implement ability (7 items), feel control (6 items), feel the resources used (14 items), and feel the threat (11 items). Each item’s ratings ranged from 1 (very weak) to 5 (very strong). Summary scores range from 48 (lowest) to 240 (highest level of health belief). The scale is widely used in China (40, 41) and in this study, the Cronbach’s α of HBS was 0.983.

#### The Chinese version of the health behavior scale for stroke patients (HBS-SP)

HBS-SP was designed by Wan et al. (42) according to the Chinese version of the Health Promotion Lifestyle Profile II. HBS-SP contains 25 items in 6 dimensions, including exercise (6 items), medication adherence (4 items), guideline adherence (6 items), nutrition (3 items), health responsibility (2 items), smoking and alcohol abstinence (2 items). The rating for each item ranges from 1 (never) to 4 (always). According to the average score of the scale, the average score for each subcategory <2 means low level health behavior, the score of 2–3 means moderate-level health behavior, and the score > 3 means high-level health behavior. This scale has good reliability and validity and can scientifically assess the health behavior level of stroke patients and high-risk populations of stroke (43, 44). In the study, the Cronbach’s α of HBS-SP was 0.867.

### Data collection

This study was conducted in an anonymous and volunteer manner. Participant recruitment took place between April to August 2023. We posted recruitment posters, which detailed the volunteer criteria and participation locations. Those willing to participate can directly go to the office alone and then researchers explain the research content to them and obtain informed consent from participants. Before starting the data collection, participants were also informed of their purpose and the choice to participate or withdraw during the experiment. The participants’ responses were filled out in questionnaires. Of the 986 questionnaires submitted, all filled out this questionnaire but 26 were excluded because they had more than 10% of missing items.

### Statistical analyses

For analysis, we used the method of data two-person input to ensure the accurate input of data. The data were analyzed using SPSS version 26.0. Normality was assessed using the Shapiro–Wilk test and normal quantile plots. The participants’ sociodemographic and disease-specific characteristics were first described using descriptive statistics. One-way analysis of variance (ANOVA) and independent sample *T*-test were used for statistical analysis. Second, Spearman correlation analysis was used to explore the correlation between knowledge, health beliefs, social support, and health behaviors. In this study, structural equation modeling (SEM) was used to test the chain mediating effect. AMOS 25.0 was used to analyze the SEM of the variables MSPSS, SKQ, HBS, and HBS-SP. The level of statistical significance was set at 0.05. To improve the accuracy of model estimation, 5,000 times Bootstrap self-sampling method was used for analysis to test the significance of the mediating effect, and 95% confidence intervals were calculated; if the 95% CI of the standardized path coefficients did not contain 0, then the intermediate effect was significant.

## Results

### Participant characteristics and analysis of group differences in MSPSS, SKQ, HBS, and HBS-SP

A total of high-risk populations of stroke (male 70.00% and female 30.00%) were recruited in the study. The average age was 62.28 ± 12.88, and 63.44% of the population were over 60. High-risk populations of stroke had a bachelor school education or above accounted for 25.52% (245/960), 94.48%(907/960) of the patients were married, and 87.08%(836/960) of the patients with a family history of stroke. The result showed that female, people with hypertension, diabetes, atrial fibrillation, and dyslipidemia had higher scores on HBS-SP (*p* < 0.05). Individuals who are current smoking, and physical inactivity had lower scores on HBS-SP (*p* < 0.05). Table 1 shows the detailed results.

**Table 1:** Participant characteristics (*N* = 960).

Variable	Frequency (*n*)	Constituent ratio (%)	MSPSS (M ± SD)	SKQ (M ± SD)	HBS (M ± SD)	HBS-SP (M ± SD)
**Age (years)**						
<40	60	6.25	59.07 ± 15.49	26.28 ± 10.68	160.52 ± 34.16	2.40 ± 0.48
40–60	291	30.31	60.98 ± 14.00	27.09 ± 9.33	161.07 ± 33.09	2.48 ± 0.40
>60	609	63.44	60.31 ± 13.41	26.4 ± 9.89	155.83 ± 34.85	2.47 ± 0.41
*F*			0.556	0.529	2.520	0.765
*p*			0.574	0.589	0.081	0.466
**Gender**						
Male	672	70.00	59.46 ± 13.84	26.39 ± 9.80	157.32 ± 35.09	2.44 ± 0.41
Female	288	30.00	62.72 ± 13.18	27.09 ± 9.69	158.32 ± 32.55	2.52 ± 0.39
*t*			−3.395	−1.028	−0.534	−2.720
*p*			0.001**	0.304	0.593	0.007**
**Education**						
Illiterate/elementary school	190	19.79	60.82 ± 13.27	26.92 ± 10.12	156.78 ± 38.49	2.46 ± 0.41
Middle school	347	36.15	60.00 ± 13.58	26.87 ± 9.66	158.86 ± 33.26	2.47 ± 0.41
High school	178	18.54	60.42 ± 15.31	26.50 ± 9.97	158.10 ± 32.68	2.49 ± 0.40
Bachelor or above	245	25.52	60.77 ± 13.10	26.04 ± 9.53	156.51 ± 33.76	2.45 ± 0.42
*F*			0.213	0.428	0.283	0.276
*p*			0.888	0.733	0.837	0.843
**Marriage status**						
Married	907	94.48	60.62 ± 13.60	26.66 ± 9.75	157.95 ± 34.08	2.47 ± 0.41
Divorced	11	1.15	58.91 ± 16.46	28.27 ± 9.98	159.09 ± 48.27	2.43 ± 0.29
Single	20	2.08	54.10 ± 15.95	22.85 ± 11.12	148.25 ± 42.77	2.27 ± 13.08
Widowed	22	2.29	59.14 ± 14.62	26.59 ± 9.22	155.73 ± 29.83	2.46 ± 0.41
*F*			1.592	1.103	0.550	1.674
*p*			0.190	0.347	0.648	0.171
**Hypertension**						
Yes	665	67.29	60.9 ± 13.69	26.67 ± 9.74	157.82 ± 34.89	2.49 ± 0.40
No	295	30.73	59.39 ± 13.76	26.45 ± 9.86	157.46 ± 33.11	2.41 ± 0.42
*t*			1.577	0.320	0.149	2.828
*p*			0.115	0.749	0.881	0.005**
**Dyslipidemia**						
Yes	411	42.81	61.02 ± 13.57	26.91 ± 9.60	162.89 ± 33.50	2.52 ± 0.41
No	549	57.19	60.00 ± 13.83	26.37 ± 9.89	153.83 ± 34.48	2.43 ± 0.40
*t*			−1.143	−0.854	−4.076	−3.634
*p*			0.253	0.394	**0.000**	**0.000**
**Diabetes**						
Yes	441	45.94	60.91 ± 13.63	26.86 ± 9.54	157.65 ± 34.09	2.50 ± 0.40
No	519	54.06	60.03 ± 13.80	26.38 ± 9.96	157.76 ± 34.58	2.44 ± 0.42
*t*			0.993	0.768	−0.049	2.048
*p*			0.321	0.443	0.961	0.041*
**Obese or overweight**						
Yes	423	44.06	61.15 ± 13.54	26.17 ± 10.21	157.24 ± 33.94	2.46 ± 0.42
No	537	55.94	59.87 ± 13.85	26.93 ± 9.40	158.08 ± 34.68	2.48 ± 0.40
*t*			−1.432	1.201	0.373	0.774
*p*			0.152	0.230	0.709	0.439
**Current smoking**						
Yes	464	48.33	60.11 ± 14.07	26.56 ± 9.97	158.79 ± 34.03	2.44 ± 0.41
No	496	51.67	60.74 ± 13.39	26.63 ± 9.59	156.70 ± 34.63	2.49 ± 0.41
*t*			0.713	0.105	−0.940	2.002
*p*			0.476	0.916	0.347	0.046*
**Physical inactivity**						
Yes	474	49.38	60.08 ± 13.54	26.18 ± 9.75	155.91 ± 33.74	2.42 ± 0.39
No	486	50.63	60.78 ± 13.90	27.01 ± 9.75	159.46 ± 34.86	2.51 ± 0.43
*t*			0.799	1.309	1.603	3.196
*p*			0.424	0.191	0.109	0.001**
**Atrial fibrillation**						
Yes	124	12.92	63.67 ± 14.79	26.73 ± 9.89	166.44 ± 32.70	2.59 ± 0.39
No	836	87.08	59.95 ± 13.50	26.58 ± 9.76	156.41 ± 34.41	2.45 ± 0.41
*t*			−2.820	−0.153	0.084	−3.616
*p*			0.005**	0.878	0.002**	**<0.001**
**Family history of stroke**						
Yes	157	16.35	59.41 ± 13.23	27.23 ± 9.21	157.46 ± 33.07	2.46 ± 0.41
No	803	83.65	60.63 ± 13.81	26.48 ± 9.87	157.76 ± 34.60	2.47 ± 0.41
*t*			1.019	−0.884	0.097	0.396
*p*			0.309	0.377	0.922	0.692

M, mean; SD, standard deviation; *t*, values of *t*-test; *F*, values of ANOVA; MSPSS, Multidimensional Scale of Perceived Social Support; SKQ, Stroke Knowledge Questionnaire; HBS, Health Beliefs Scale; HBS-SP, Health behavior scale for stroke patients.*p < 0.05;**p < 0.01;***p < 0.001. Bold values means *p* < 0.05.

### Correlation of MSPSS, SKQ, HBS, and HBS-SP

Table 2 showed that total or average scores for MSPSS, SKQ, HBS, and HBS-SP were 60.64 ± 13.72, 26.60 ± 9.77, 157.71 ± 34.34, and 2.46 ± 0.41, respectively. Table 2 also presented the statistical results of correlation coefficients that the HBS was obviously and positively correlated to MSPSS (*r* = 0.300, *p* < 0.01) and SKQ (*r* = 0.166, *p* < 0.01). In addition, HBS-SP was obviously and positively related to MSPSS (*r* = 0.336, *p* < 0.01), SKQ (*r* = 0.355, *p* < 0.01), and HBS (*r* = 0.519, *p* < 0.01).

**Table 2:** Spearman correlation analysis of each variable (*N* = 960).

Variable	M ± SD	1	2	3	4	5	6	7	8	9	10	11	12	13	14	15	16	17	18	19	20	21	22	23	24
1. MSPSS	60.64 ± 13.72	1	–	–	–	–	–	–	–	–	–	–	–	–	–	–	–	–	–	–	–	–	–	–	–
2. Friends support	20.83 ± 4.91	0.853^**^	1	–	–	–	–	–	–	–	–	–	–	–	–	–	–	–	–	–	–	–	–	–	–
3. Family support	19.67 ± 4.74	0.941^**^	0.730^**^	1	–	–	–	–	–	–	–	–	–	–	–	–	–	–	–	–	–	–	–	–	–
4. Significant other support	19.93 ± 4.69	0.979^**^	0.820^**^	0.893^**^	1	–	–	–	–	–	–	–	–	–	–	–	–	–	–	–	–	–	–	–	–
5. SKQ	26.60 ± 9.77	0.099^**^	0.091^**^	0.079*	0.103^**^	1	–	–	–	–	–	–	–	–	–	–	–	–	–	–	–	–	–	–	–
6. Premonitory symptoms	3.71 ± 3.01	0.006	0.003	−0.007	0.011	0.727^**^	1	–	–	–	–	–	–	–	–	–	–	–	–	–	–	–	–	–	–
7. First aid treatment	2.51 ± 1.49	0.017	0.006	0.01	0.024	0.705^**^	0.448^**^	1	–	–	–	–	–	–	–	–	–	–	–	–	–	–	–	–	–
8. Risk factors	8.17 ± 3.82	0.076*	0.067*	0.065*	0.076*	0.875^**^	0.503^**^	0.586^**^	1	–	–	–	–	–	–	–	–	–	–	–	–	–	–	–	–
9. Safety of medication	2.30 ± 1.05	0.110^**^	0.100^**^	0.089^**^	0.107^**^	0.367^**^	0.165^**^	0.235^**^	0.243^**^	1	–	–	–	–	–	–	–	–	–	–	–	–	–	–	–
10. Mode of behavior	8.12 ± 2.70	0.166^**^	0.171^**^	0.142^**^	0.165^**^	0.711^**^	0.315^**^	0.368^**^	0.513^**^	0.182^**^	1	–	–	–	–	–	–	–	–	–	–	–	–	–	–
11. Rehabilitation	1.79 ± 1.28	0.058	0.035	0.041	0.070*	0.682^**^	0.376^**^	0.447^**^	0.548^**^	0.210^**^	0.470^**^	1	–	–	–	–	–	–	–	–	–	–	–	–	–
12. HBS	157.71 ± 34.34	0.300^**^	0.259^**^	0.284^**^	0.289^**^	0.166^**^	0.106^**^	0.077*	0.128^**^	0.133^**^	0.136^**^	0.149^**^	1	–	–	–	–	–	–	–	–	–	–	–	–
13. Personal health belief	33.28 ± 9.09	0.288^**^	0.245^**^	0.269^**^	0.278^**^	0.190^**^	0.122^**^	0.095^**^	0.139^**^	0.140^**^	0.164^**^	0.178^**^	0.899^**^	1	–	–	–	–	–	–	–	–	–	–	–
14. Feel the implement ability	22.49 ± 5.80	0.284^**^	0.248^**^	0.272^**^	0.277^**^	0.171^**^	0.122^**^	0.093^**^	0.128^**^	0.127^**^	0.129^**^	0.151^**^	0.904^**^	0.848^**^	1	–	–	–	–	–	–	–	–	–	–
15. Feel control	19.75 ± 5.11	0.295^**^	0.257^**^	0.283^**^	0.284^**^	0.173^**^	0.122^**^	0.087^**^	0.133^**^	0.108^**^	0.140^**^	0.151^**^	0.906^**^	0.813^**^	0.853^**^	1	–	–	–	–	–	–	–	–	–
16. Feel the resources use	46.61 ± 11.08	0.307^**^	0.263^**^	0.289^**^	0.299^**^	0.144^**^	0.092^**^	0.076*	0.115^**^	0.093^**^	0.118^**^	0.124^**^	0.897^**^	0.742^**^	0.788^**^	0.804^**^	1	–	–	–	–	–	–	–	–
17. Feel the threat	35.58 ± 8.96	0.127^**^	0.113^**^	0.118^**^	0.114^**^	0.057	0.021	−0.004	0.05	0.110^**^	0.046	0.053	0.711^**^	0.500^**^	0.496^**^	0.532^**^	0.480^**^	1	–	–	–	–	–	–	–
18. HBS-SP	61.68 ± 10.23	0.336^**^	0.289^**^	0.312^**^	0.330^**^	0.355^**^	0.221^**^	0.274^**^	0.267^**^	0.209^**^	0.286^**^	0.297^**^	0.519^**^	0.499^**^	0.525^**^	0.504^**^	0.472^**^	0.274^**^	1	–	–	–	–	–	–
19. Exercise	12.89 ± 4.61	0.166^**^	0.130^**^	0.164^**^	0.156^**^	0.182^**^	0.113^**^	0.151^**^	0.137^**^	0.059	0.150^**^	0.173^**^	0.281^**^	0.240^**^	0.292^**^	0.292^**^	0.244^**^	0.177^**^	0.465^**^	1	–	–	–	–	–
20. Nutrition	13.77 ± 4.45	0.181^**^	0.130^**^	0.186^**^	0.185^**^	0.150^**^	0.093^**^	0.127^**^	0.111^**^	0.131^**^	0.109^**^	0.111^**^	0.205^**^	0.202^**^	0.222^**^	0.179^**^	0.215^**^	0.070*	0.539^**^	−0.074*	1	–	–	–	–
21. Medication adherence	13.27 ± 2.65	0.264^**^	0.229^**^	0.236^**^	0.273^**^	0.251^**^	0.169^**^	0.154^**^	0.208^**^	0.137^**^	0.192^**^	0.197^**^	0.317^**^	0.295^**^	0.306^**^	0.305^**^	0.285^**^	0.190^**^	0.585^**^	0.217^**^	0.284^**^	1	–	–	–
22. Guideline adherence	9.86 ± 2.64	0.250^**^	0.212^**^	0.236^**^	0.243^**^	0.264^**^	0.159^**^	0.223^**^	0.206^**^	0.132^**^	0.220^**^	0.194^**^	0.390^**^	0.387^**^	0.375^**^	0.362^**^	0.354^**^	0.213^**^	0.681^**^	0.219^**^	0.393^**^	0.399^**^	1	–	–
23. Health responsibility	5.53 ± 2.09	0.169^**^	0.130^**^	0.168^**^	0.172^**^	0.258^**^	0.175^**^	0.189^**^	0.195^**^	0.152^**^	0.191^**^	0.229^**^	0.222^**^	0.194^**^	0.253^**^	0.217^**^	0.201^**^	0.117^**^	0.646^**^	0.334^**^	0.382^**^	0.352^**^	0.368^**^	1	–
24. Smoking and alcohol abstinence	6.35 ± 1.88	0.209^**^	0.182^**^	0.193^**^	0.209^**^	0.179^**^	0.081*	0.153^**^	0.118^**^	0.106^**^	0.184^**^	0.168^**^	0.320^**^	0.296^**^	0.317^**^	0.294^**^	0.291^**^	0.192^**^	0.635^**^	0.236^**^	0.344^**^	0.425^**^	0.421^**^	0.409^**^	1

*p < 0.05;**p < 0.01;***p < 0.001.

### Mediations of MSPSS, SKQ, HBS, and HBS-SP

**Figure 2:**
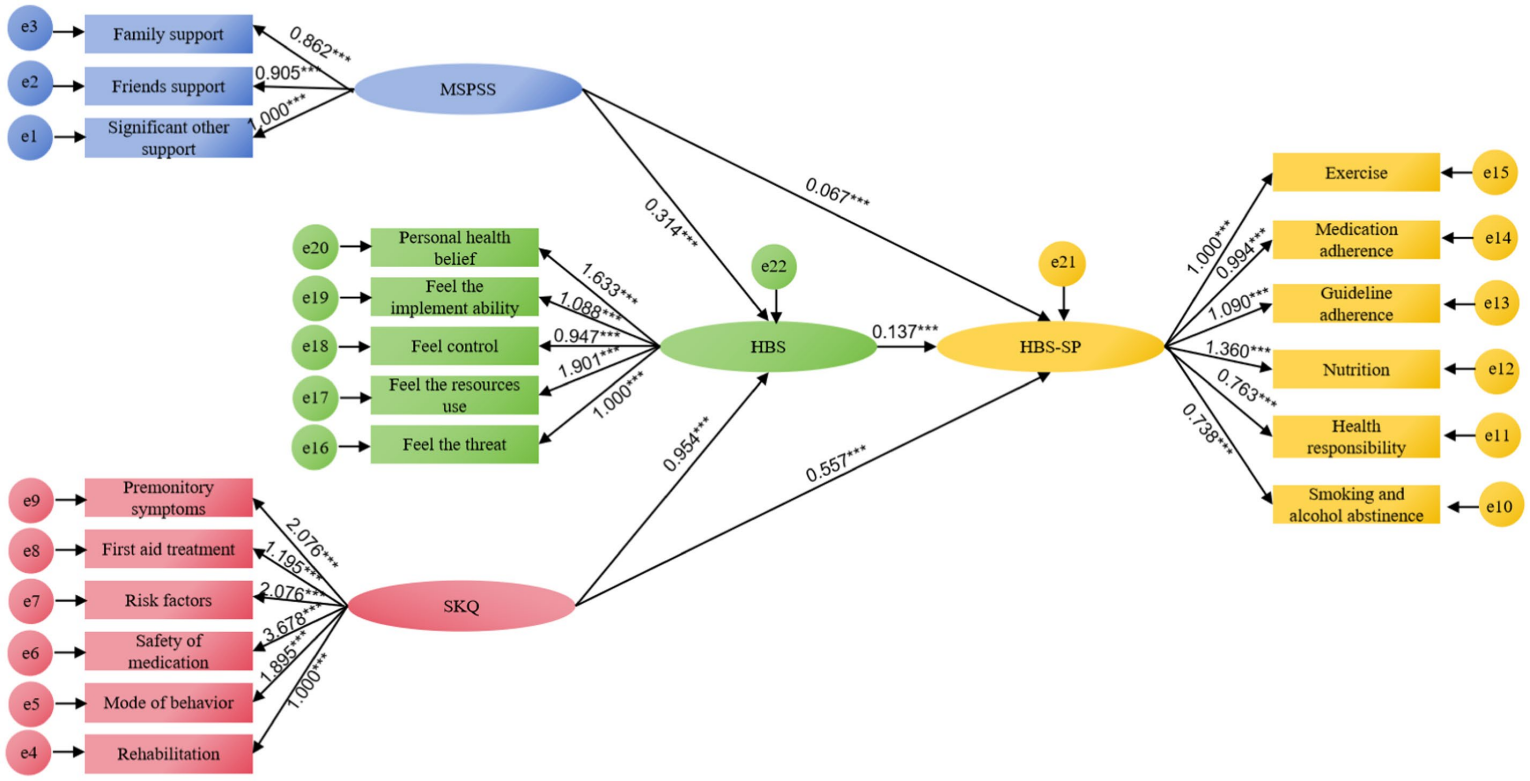
Results of pathway analysis of MSPSS, SKQ, HBS, and HBS-SP. ***p < 0.001.

To test the mediating role of HBS between the independent variables MSPSS, and SKQ, and after the modification of the model, the fitting index results were obtained: X2/df = 2.724 < 5.000, NFI = 0.936 > 0.900, GFI = 0.946 > 0.900, TLI = 0. 954 > 0.900, RFI = 0.928 > 0.900, CFI = 0.959 > 0.900, and RMSEA = 0.042 < 0.080, the overall model fitted well. As shown in Figure 2, all standardized path coefficients were meaningful (*p* < 0.05). MSPSS had a meaningful positive predictive effect on HBS and HBS-SP (β = 0.314, *p* < 0.001; β = 0.067, *p* < 0.001), and SKQ had a meaningful positive predictive effect on HBS and HBS-SP (β = 0.954, *p* < 0.001; β = 0.557, *p* < 0.001).

From Table 3, the 95% CI of the above four paths does not contain 0, which suggested that the total effect, direct effect, and total indirect effect in the model are statistically significant. Through further analysis of the effects of variables, it was found that the total effect of MSPSS to HBS-SP was 0.110, the direct effect was 0.067 (effect size 8.40%), the indirect effect was 0.043 (effect size 5.39%), the total effect of SKQ to HBS-SP was 0.688, the direct effect was 0.557 (effect size 69.80%), the indirect effect was 0.131 (effect size 16.42%), and the total effect was 0.798, the direct effect was 0.624 (effect size 8.40%), the indirect effect was 0.174 (effect size 5.39%).

**Table 3:** Mediating effect test between MSPSS, SKQ, HBS, and HBS-SP.

Paths	Bootstrapping			95%Bias-corrected CI		Relative intermediary effect (%)
	Effect value	Boot S.E.	*p*	Boot LLCI	Boot ULCI	
MSPSS→ HBS-SP	0.067	0.015	<0.001	0.231	0.363	8.40
SKQ → HBS-SP	0.557	0.097	<0.001	0.098	0.236	69.80
MSPSS→HBS → HBS-SP	0.043	0.009	<0.001	0.028	0.062	5.39
SKQ → HBS → HBS-SP	0.131	0.034	<0.001	0.074	0.212	16.42
Total effect between MSPSS and HBS-SP	0.110	0.112	<0.001	0.040	0.100	13.78
The total effect between SKQ and HBS-SP	0.688	0.020	<0.001	0.387	0.769	86.22
Total effect	0.798	0.221	<0.001	0.566	1.053	100.00

## Discussion

Previous studies have shown that having unhealthy behavior plays a critical role in the onset and recurrence of stroke (28, 45). The COM-B model is used in the study to explore the influencing factors of health behavior and the mediating function of health beliefs in populations at high risk for stroke. This is the first study that, to our knowledge, highlights the significance of health beliefs as a medium between knowledge, social support, and health behaviors among individuals at high risk for stroke. In line with earlier research (42, 44, 46, 47), we discovered that high-risk groups’ preventive behavior against stroke was at a moderate level. We also discovered a statistically significant beneficial relationship between knowledge, social support, health beliefs, and health behaviors. In addition, the connection between knowledge, social support, and health behaviors was mediated by health attitudes.

It should be highlighted that hypertension (67.29%) was the most prevalent risk factor among high-risk groups in our investigation, which has been supported by numerous other experimental studies (2, 48–50). Physical inactivity and current smoking are the second and third risk factors, respectively, which are preventable risk factors regarding lifestyle. Given these preventable and controllable risk factors, it is suggested that the specific evaluation of health behavior be done rapidly and efficiently. Interventions like health education that promote awareness of medical problems and encourage the improvement of health behavior are also suggested. In addition, the examination of discrepancies in HBS-SP among different demographic characteristics has proved that males have better health behavior than females. Those with chronic diseases (like hypertension or diabetes) are more likely to have healthier lifestyles, which is consistent with previous studies (51).

In this study, we identified a significant positive association between stroke knowledge, social support, health beliefs, and health behavior among high-risk groups in China, which supports H1. Knowledge is the foundation to change behavior and this conclusion is consistent with previous research findings on their relationships. For example, in 2008, using data from a survey of diabetes patients in China, researchers examined the knowledge of diabetic foot prevention and nursing practice (52). They found patients with higher knowledge scores were more effective in practice. Researchers also have verified the role of health education in enhancing behavior beliefs in another study (53). Besides, patients usually can not be treated continuously because of poor support according to previous research (54), which suggests adequate social support has a positive impact on patients and helps them develop healthy behaviors. In addition, health beliefs were directly associated with health behavior. The finding is consistent with previous studies (55, 56). According to the health beliefs model (HBM), one of the well-known theories of health behavior, beliefs in health risk predict the likelihood of engaging in health behavior (57, 58). Beliefs are a key concept in understanding health-related cognition and behavior. Thus, patients with high-level health beliefs, such as higher levels of self-efficacy and confidence, motivation to take action, stronger ability to cope with and adapt to adversity, and accurate risk perception, will have higher levels of healthy behaviors (e.g., diet, physical activity, and treatment adherence) (59). Peng et al. (60) also investigated the relationship between health beliefs and health behaviors among stroke patients in China, which proved that higher beliefs mean better health behaviors.

In the present study, health beliefs function as a significant mediator between social support, stroke knowledge and health behaviors. That is high-risk groups’ beliefs in their skill to prevent stroke is an important mechanism linking knowledge and social support with health behaviors. Therefore, H2 is supported. Health beliefs are an attitude toward health and disease. People with strong health beliefs believe in their ability to maintain or regain their health and believe that they can better prevent disease (e.g., strokes) by adhering to certain behaviors (e.g., taking medication regularly) or making certain changes (e.g., quitting smoking, losing weight). Although no direct studies currently focus on the mediator role of health beliefs between the above factors, studies have explored the relationship between health behavior and them (61). Therefore, the government should focus on educating the high-risk groups on stroke and its risk factors since this will help them understand the disease, thereby encouraging the building of good beliefs and finally enhancing health behaviors.

## Limitations

There are several restrictions on this study. First, this study was cross-sectional in design, and therefore, any causal relationship could not be established and the dynamic changes in factors associated with health-related behaviors were not well understood. Thus, in the future experimental and longitudinal research are necessary. Second, the sample was drawn from a single physical examination center. The sample of individuals might not accurately and adequately represent the population. Third, this study recruited participants on a voluntary method, which may introduce a certain bias as individuals willing to participate may have better knowledge and behaviors related to the study. Furthermore, participants’ occupations and whether they have been involved in other health education programs were not taken into account. In future research, it is advisable to collect demographic information to minimize bias. In addition, the overall study population included more men (70%) than women (30%), which might cause bias in the results.

## Conclusion

The study’s findings show that knowledge, social support, and health beliefs significantly and positively correlate with inadequate health behaviors reported by high-risk individuals for stroke. In addition, the link between knowledge, social support, and health behaviors is mediated by health beliefs. These results imply that regular evaluation of health behaviors, knowledge, health beliefs, and social support, as well as the use of targeted interventions, are crucial for lowering the risk of stroke. Future studies are necessary to explore these therapies’ effectiveness using longitudinal data based on this study.

## Data availability statement

The original contributions presented in the study are included in the article, further inquiries can be directed to the corresponding authors.

## Ethics statement

The studies involving humans were approved by Ethics Committee of Shanghai Changhai Hospital. The studies were conducted in accordance with the local legislation and institutional requirements. The participants provided their written informed consent to participate in this study.

## Author contributions

MC: Conceptualization, Formal analysis, Investigation, Project administration, Supervision, Writing – original draft, Writing – review & editing. MW: Investigation, Methodology, Writing – original draft. MQ: Investigation, Methodology, Validation, Writing – original draft. XH: Investigation, Writing – original draft. DL: Data curation, Investigation, Writing – original draft. LY: Data curation, Investigation, Writing – original draft. LG: Data curation, Investigation, Writing – original draft. WC: Project administration, Supervision, Writing – original draft. YW: Investigation, Supervision, Writing – original draft. JZ: Investigation, Writing – original draft. BY: Investigation, Writing – original draft. JL: Project administration, Supervision, Writing – review & editing. LZ: Project administration, Supervision, Writing – review & editing.
